# Cortical connectomic mediations on gamma band synchronization in schizophrenia

**DOI:** 10.1038/s41398-022-02300-6

**Published:** 2023-01-19

**Authors:** Xiaoming Du, Stephanie Hare, Ann Summerfelt, Bhim M. Adhikari, Laura Garcia, Wyatt Marshall, Peng Zan, Mark Kvarta, Eric Goldwaser, Heather Bruce, Si Gao, Hemalatha Sampath, Peter Kochunov, Jonathan Z. Simon, L. Elliot Hong

**Affiliations:** 1 Maryland Psychiatric Research Center, Department of Psychiatry, University of Maryland School of Medicine, Baltimore, MD USA; 2 Department of Electrical & Computer Engineering, University of Maryland, College Park, MD USA; 3 Department of Biology, University of Maryland, College Park, MD USA; 4 Institute for Systems Research, University of Maryland, College Park, MD USA

**Keywords:** Schizophrenia, Diagnostic markers

## Abstract

Aberrant gamma frequency neural oscillations in schizophrenia have been well demonstrated using auditory steady-state responses (ASSR). However, the neural circuits underlying 40 Hz ASSR deficits in schizophrenia remain poorly understood. Sixty-six patients with schizophrenia spectrum disorders and 85 age- and gender-matched healthy controls completed one electroencephalography session measuring 40 Hz ASSR and one imaging session for resting-state functional connectivity (rsFC) assessments. The associations between the normalized power of 40 Hz ASSR and rsFC were assessed via linear regression and mediation models. We found that rsFC among auditory, precentral, postcentral, and prefrontal cortices were positively associated with 40 Hz ASSR in patients and controls separately and in the combined sample. The mediation analysis further confirmed that the deficit of gamma band ASSR in schizophrenia was nearly fully mediated by three of the rsFC circuits between right superior temporal gyrus—left medial prefrontal cortex (MPFC), left MPFC—left postcentral gyrus (PoG), and left precentral gyrus—right PoG. Gamma-band ASSR deficits in schizophrenia may be associated with deficient circuitry level connectivity to support gamma frequency synchronization. Correcting gamma band deficits in schizophrenia may require corrective interventions to normalize these aberrant networks.

## Introduction

Cortical gamma band (∼40 Hz) neural oscillations play a pivotal role in integrating sensory information across distributed cortical areas [1]. The involvement of gamma-band oscillations in multi-modal cognitive activities has been suggested in spatiotemporal integration of perception [1], speech [2], associative learning [3], visual attention [4], and feature binding [5]. Abnormal gamma band oscillation has been hypothesized to be related to cognitive deficits in these areas, many of which are known to be significantly impaired in schizophrenia. The auditory steady-state response (ASSR) entrains neural oscillations in the brain to a specific frequency of auditory stimuli and has been used to assess the integrity of cortical oscillatory activity [6–8]. Reduced gamma band ASSR has been consistently observed in patients with schizophrenia [6, 9–11] and replicated in EEG [10, 12–24] and MEG studies [25–30], observed not just in chronic schizophrenia, but also in first-episode psychosis [14, 22], the ultra-high risk for psychosis [22], and non-ill first degree relatives of the patients [10]. Although there are also studies showing null or even reversed findings in patients [11, 31–34], it is one of the more robust electrophysiological biomarkers in schizophrenia. Correcting gamma band deficits have been argued to be important for developing more effective treatment for cognitive deficits in schizophrenia [23, 35], which may, in part, rely on a better understanding of the underlying brain circuitry.

The ASSR generators are believed to be at the primary auditory cortex or the superior temporal plane [8, 36–39] based on MEG source localization studies [27, 38–44]. EEG dipole modeling supported similar sources in the bilateral auditory cortices [43]. In a functional magnetic resonance imaging (fMRI) study, Heschl’s gyrus, along with the medial geniculate body and inferior colliculus, were found to be associated with 40-Hz amplitude-modulated tones [45]. Positron emission tomography (PET) studies also suggest that activation of bilateral auditory cortices may be associated with 40-Hz ASSR [46]. The 40 Hz ASSR deficits in schizophrenia have been localized to the superior temporal plane [20] and primary auditory cortex [27, 28] with dipole models or by associations to the reduced cortical volume of the superior temporal gyrus [34], although it remains unknown whether dysregulated auditory cortex activity fully explains 40 Hz ASSR deficits in schizophrenia.

The 40 Hz ASSR is acquired using continuous stimulations over a long duration (typically around 300 to 500 ms), which would likely engage the extensive brain networks known to be associated with the primary auditory cortex [47–50]. We propose that a deficit in the underlying functional network of the primary auditory cortex may lead to the inability to sustain synchronization of the gamma band, contributing to the impaired 40 Hz ASSR in schizophrenia. To examine the functional connections between brain regions that may account for the gamma band ASSR deficit in schizophrenia, resting-state functional connectivity (rsFC) was obtained using the left and right primary auditory cortex as the initial seeds to identify rsFC that are associated with 40 Hz ASSR in patients with schizophrenia and healthy individuals. We then tested whether the gamma band ASSR deficit in schizophrenia was mediated by those functional connections with a mediation analysis. Revealing the neural underpinnings of this gamma band ASSR deficit could shed light on the mechanism of auditory-related gamma synchronization abnormalities in schizophrenia.

## Materials and methods

### Participants

The study included 66 patients with schizophrenia (*n* = 62) or schizoaffective disorder (*n* = 4) (referred together as schizophrenia for brevity) and 85 healthy individuals (Table 1). Patients were recruited from the Maryland Psychiatric Research Center and neighboring mental health clinics in the Baltimore area. Controls were recruited from local media advertisements. The Structured Clinical Interview for DSM-IV was used to confirm the diagnoses in patients and the absence of current DSM-IV Axis I diagnoses in healthy controls. Exclusion criteria were major medical and neurological illnesses, head injury, and substance dependence or substance abuse (except nicotine). Eight schizophrenia patients were not on antipsychotic medications, 51 were atypical, and 12 were on typical, including five on both atypical and typical antipsychotics (Table 1). No patients took benzodiazepines at the time of scanning. All subjects gave their written informed consent approved by the local Institutional Review Board. The ASSR EEG data and resting-state functional MRI data were collected in separate sessions. There was no significant group difference in intervals between the two sessions, although it was longer in the patient group (median intervals for schizophrenia and healthy individuals were 10 and 4 weeks, respectively, 95% CI (−25.4 – 0.3), *p* > 0.05). The current sample of patients and controls (66 and 85, respectively) was a subset of the previously reported sample (128 and 108, respectively) on 40 Hz ASSR [10] who had also completed fMRI; the fMRI-ASSR data are not previously reported.

**Table 1:** Demographic and clinical characteristics.

	Schizophrenia *n* = 66	Healthy control *n* = 85	*t*, *F*, or *χ*^2^	*p*
Age (years) (±SD)	33.92 ± 12.80	35.58 ± 13.98	0.75	0.46
Male/Female	44/22	51/34	0.71	0.40
BPRS total score	40.03 ± 10.99	-	-	-
BNSS total score	18.82 ± 15.19	-	-	-
Education (years)	12.97 ± 2.49	14.25 ± 2.48	3.13	0.002
Smoker/non-smoker	23/43	21/64	1.85	0.17
Antipsychotic medication				
Typical	12^a^	-	-	-
Atypical	51^a^	-	-	-
Medication-free	8	-	-	-
CPZ (mg)	573 ± 548	-	-	-
GAF total score	168.62 ± 41.36	254.20 ± 40.74	12.31	<0.0001
40 Hz ASSR power ^b^	76.23 ± 12.70	81.52 ± 12.14	2.69	0.008

*BPRS* brief psychiatric rating scale, *BNSS* brief negative symptom scale, *CPZ* chlorpromazine equivalent of medication dose, *GAF* global assessment of functioning, *SD* standard deviation.

^a^Five patients took both typical and atypical antipsychotic medications.

^b^ASSR were presented as normalized ASSR power [10].

### ASSR paradigm

The details of the ASSR paradigm have been reported elsewhere [10, 11]. Briefly, trains of click sounds at 72 dB and of 1 ms duration were delivered via headphones at 40 Hz. Each train consisted of 15 clicks that last for 375 ms. There were 75 stimulus trains (trials) with 750 ms intervals between the end of a train and the beginning of the next. The total durations were 1 min and 25 s.

Electroencephalography (EEG) recording was performed in a sound-attenuated chamber using a 64-channel Quick-Cap with sintered silver/silver-chloride (Ag/AgCl) electrodes and a Neuroscan SynAmp2 amplifier (Compumedics, Charlotte, NC). The EEG data were recorded at a sampling rate of 1000 Hz with a 0.1–200 Hz bandpass filter. Impedance was kept below 5 kΩ. Linked mastoid electrodes served as the reference. The EEG data were re-referenced to average reference, high-pass filtered at 0.8 Hz, and detrended during offline analysis. Ocular artifacts were removed using the time-shift-PCA algorithm [51], with ocular channels as references. Participants were instructed to relax, remain alert, and keep eyes open during the recording.

### Normalized ASSR power

Rather than using individual channels (e.g., CZ or FZ), we adapted the denoising source separation (DSS) algorithm to maximize ASSR response reliability, where individual EEG channels are spatially combined [10, 52–54]. DSS is specifically designed for use with data from multi-trial evoked responses or narrowband signals and works by enhancing stimulus-driven activity over stimulus-unrelated activity, with its components ordered according to their reliability [52–54]. Raw 40 Hz ASSR power was obtained at the 40 Hz frequency and background power was calculated by averaging spectral power over 1 Hz width frequency bands (on either side of the 40 Hz frequency, after leaving a guard band of 0.5 Hz on either side). Normalized 40 Hz ASSR power was then calculated as the ratio of raw ASSR power and respective background power as in our previous studies [10, 53]. This normalization with respect to background power remarkably reduces subject-to-subject variability of frequency response profiles [55].

### Assessments of symptom and function

The 20-item Brief Psychiatric Rating Scale (BPRS) was administered to patients for assessing their overall clinical symptoms [56]. Positive symptoms were obtained by the summation of sub-items (i.e., item 4, 7, 8, 11, 12, 15, and 20) of the 20-item version of the BPRS. Negative symptoms were assessed by using the Brief Negative Symptom Scale (BNSS), a 13-item clinician-rated scale validated for the assessment of negative symptoms in schizophrenia patients in the following areas: blunted affect, alogia, asociality, anhedonia, and avolition [57]. All BPRS and BNSS raters were formally trained until raters achieved acceptable reliability. The MIRECC version of the global assessment of functioning (GAF) was also adopted for measuring global functioning [58]. The total scores were obtained from each of the three assessments (i.e., BPRS, BNSS, and GAF).

### Imaging data acquisition

All imaging was performed using a Siemens 3 T Trio MRI system (Erlangen, Germany) equipped with a 32-channel head coil. High-resolution T1-weighted magnetization prepared rapid acquisition gradient-echo images (MPRAGE) were acquired (repetition time (TR)/echo time (TE) 2200/2.81 ms, flip angle = 13°, field of view (FOV) = 256 mm, 0.8 mm^3^ spatial resolution). Resting-state functional T2*-weighted images were obtained using a single-shot gradient-recalled, echo-planar pulse sequence (TR/TE = 2 s/27 ms; flip angle = 90°; FOV = 220 mm; 128 × 128 matrix; 1.72 mm^2^ in-plane resolution; 4 mm slice thickness; 37 axial slices, 15 min scan for 444 volumes). Participants were asked to keep their eyes closed, relax, and not to think about anything in particular. Post-scan questions confirmed that participants did not fall asleep during the scan.

### Imaging data preprocessing and analysis

Standard resting-state functional MRI data processing was carried out using Analysis of Functional NeuroImages (AFNI) [59] software (Version AFNI_18.2.15). We used the afni_proc.py script for preprocessing and @ANATICOR script for noise detection and removal [60]. The first four volumes of the functional data were removed to ensure magnetization steady-state in the remaining volumes, which were de-spiked and slice-time corrected. The preprocessed data were spatially smoothed to a full width at half maximum (FWHM) of 4 mm. The linear trend, six motion parameters (three rotational and three translational directions), their six temporal derivatives (rate of change in rotational and translational motion), and time courses from the white matter and cerebrospinal fluid (CSF) were regressors of no interest. Time points with excessive motion (>0.2 mm) and their neighboring time points were censored from statistical analysis. There was no significant difference in head motion between the two groups (*t*(149) = −0.27, *p* = 0.79). For group analysis, images were spatially normalized to a standard space and the significant clusters were automatically labeled according to Talairach-Tournoux Atlas distributed within AFNI [61].

Individual statistical maps were then calculated using seed-based correlation analysis to infer the functional connectivity of the seed with the rest of the brain. Two a priori seed-regions of interest (ROIs) were selected: left (Talairach coordinates x, y, z = −53, −16, 3) and right (x, y, z = 55, −16, 6) superior temporal gyrus (STG) [62]. Two spheres with a 10 mm radius were placed on each subject’s structural images centered at left and right STG, respectively. The two coordinates at STG were derived from previous functional imaging data [62] so the ROI spheres did not cover the whole STG but only a small portion of the STG regions centered at the peak coordinates of the auditory fMRI-derived STG. White matter and CSF were removed from the seed ROI using the masks obtained from FreeSurfer in the preprocessing of functional data [63]. Then, the correlations between the mean time series within seed ROI and the time series of each voxel in the brain were obtained for each participant. Pearson’s correlation coefficients were converted to *z* values using Fisher’s *r*-to-*z* transform. Volumetric reductions and cortical thinning of gray matter in schizophrenia have been demonstrated in temporal regions [64–66]. However, the gray matter volumes within seed ROIs that covered the partial region of STG did not significantly differ between the two groups (left STG: *t*(149) = 0.26, *p* = 0.80; right STG: *t*(149) = −1.32, *p* = 0.19), although this was based on a small spherical area of the STG defined for fMRI analysis purpose, as STG volume reduction is one of the most replicated findings in schizophrenia [64–66].

To confirm the robustness of the observed rsFC linked to a 40 Hz ASSR deficit in schizophrenia, the destination node of the rsFC was used as the seed to repeat the above-mentioned analyses. This was used to evaluate (1) whether the observed STG-seeded rsFC, which was related to 40 Hz ASSR deficit, can be replicated when the destination node was utilized as the seed; (2) whether there was other 40 Hz ASSR-related rsFC, which was not originated from primary auditory cortex.

### Statistical analysis

The main goal of the study was to examine what were the rsFC correlates with 40 Hz ASSR deficits in schizophrenia. First, we confirmed the 40 Hz ASSR deficit in schizophrenia with the current sample by comparing patients with schizophrenia and healthy controls (Table 1). Second, for voxel-wise group analysis, multiple linear regression was applied across the whole brain for 40 Hz ASSR (AFNI 3dMVM function) [67]. The dependent variable was whole-brain voxel-wise rsFC with left or right STG as seed and the independent variables were ASSR, Group (schizophrenia and controls), ASSR × Group, and age. Candidate rsFC related to 40 Hz ASSR deficit in schizophrenia were either (1) a significant ASSR×Group interaction, which would imply different rsFC-ASSR relationships in the two groups; or (2) a significant positive (or negative) rsFC-ASSR association in both patients and controls alike but the rsFC were significantly weaker (stronger) in patients with schizophrenia. The significant clusters were determined by estimating the cluster-size threshold with a voxel-wise height threshold (*p* < 0.001) using the updated 3dClustSim with the spatial autocorrelation function [68–70] to obtain corrected *p* < 0.05. For rsFC showing a significant main effect of 40 Hz ASSR or a significant 40 Hz ASSR × Group interaction, we examined if there was a significant difference in rsFC between the two groups. Further, we tested the associations between ASSR and rsFC in each group to explore if the rsFC-ASSR association demonstrated from the ASSR main effect was also exhibited in each group or revealed the simple effect of the ASSR×Group interaction. The false-discovery rate (FDR) approach was used for controlling multiple comparison issues caused by multiple significant clusters [71]. The rsFC thus identified were further used to test for potential contributions to clinical symptoms using linear regressions or the Pearson correlation method. The demographic data were compared using independent-sample *t*-tests for continuous values and the Chi-squared test for categorical values.

Lastly, to test whether the difference of 40 Hz ASSR between patients with schizophrenia and healthy individuals was mediated by these rsFC, the total effects, direct effects, and indirect effects of diagnosis on 40 Hz ASSR were evaluated with these rsFC as the mediators and age as the covariate, using mediation analysis method from PROCESS macro for SPSS [72]. The bootstrap method was used for estimating a 95% confidence interval (CI) with 10,000 resamples.

## Results

There was no significant difference between the groups in age, gender, or smoking status, but patients showed significantly less formal education completed and global functioning compared with healthy controls (Table 1). Adding gender [18, 73] and smoking status as additional covariates showed similar findings and those were not reported below.

### STG-seeded functional connectivity underlying gamma ASSR

**Fig. 1:**
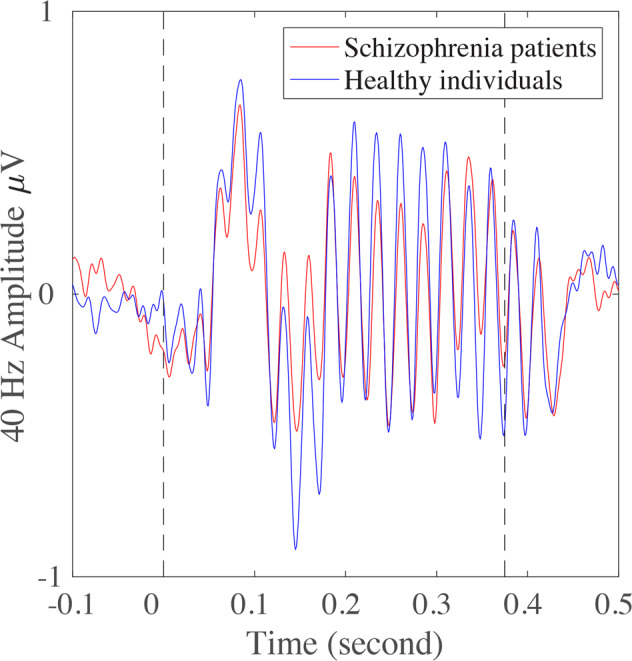
Grand averages of 40 Hz auditory steady-state response (ASSR). The ASSRs for both schizophrenia patients and healthy individuals are in red and blue, respectively.

**Fig. 2:**
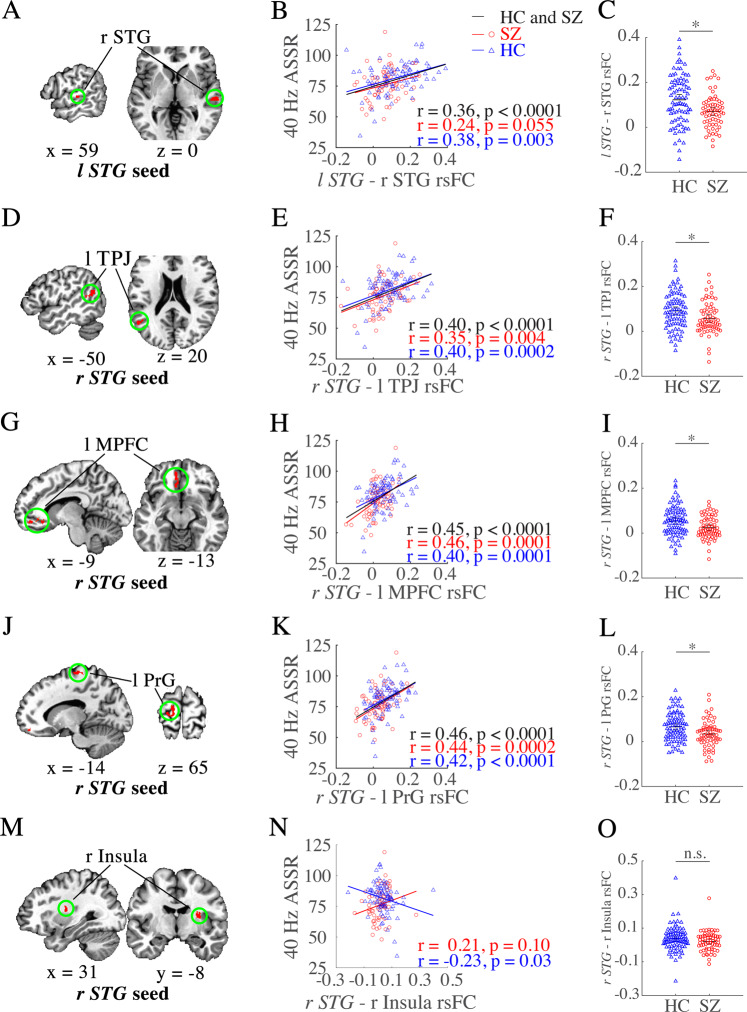
Five brain regions whose resting-state functional connectivity (rsFC) with superior temporal gyrus (STG) were associated with 40 Hz auditory steady-state response (ASSR). The five brain regions are shown in five sets of figures (**A**, **D**, **G**, **J**, **M**) on the left column and each region is shown by sagittal and axial views. **A** is from the left *STG* seed. The remaining **D**–**M** are from the right *STG* seed. **A** Left *STG*-seeded rsFC with right STG was significantly and positively linked to 40 Hz ASSR in the combined groups and within each group (**B**); and was significantly weaker in schizophrenia patients as compared with healthy individuals (**C**). **D**–**O**: Right *STG*-seeded rsFC with left temporoparietal junction (TPJ) (**D**), left medial prefrontal cortex (MPFC) (**G**), and left precentral gyrus (PrG) (**J**) also significantly predicted 40 Hz ASSR in both combined groups and within each group (**E**, **H**, **K**), and patients with schizophrenia showed significantly weaker rsFC (**F**, **I**, **L**). The only rsFC × ASSR interaction was found at right *STG*—right insula rsFC (**M**, **N**), but this rsFC was not significantly different between the two diagnostic groups (**O**). Seed region was written in italic. * FDR corrected *p* < 0.05 for five comparisons. n.s. non-significant. The left is shown on the left side of the MRI image.

Schizophrenia patients showed significantly reduced gamma (40 Hz) synchronizations compared to controls (*p* = 0.008) (Fig. 1 and Table 1). There were four significant main effects of 40 Hz ASSR at rsFC: (1) between *left STG*—right STG; (2) *right STG*—left temporoparietal junction (TPJ) (Fig. 2A, D; all statistical details also in Table 2); (3) between *right STG*—left medial prefrontal cortex (MPFC); and (4) between *right STG*—left precentral gyrus (PrG) (Fig. 2G, J). These positive associations between rsFC and 40 Hz ASSR across groups were also significant in patients and controls independently in most rsFC (except *left STG*—right STG rsFC, which was only present in the schizophrenia group) (Fig. 2B, E, H, K). The comparisons of the correlation coefficients among these rsFC showed that none of them was significantly different between the diagnostic groups (*z* = 0.14 to 0.91, *p* = 0.37–0.89). Meanwhile, schizophrenia patients had significantly weaker rsFC as compared with healthy individuals at all these rsFC (Fig. 2C, F, I, L). These findings suggest that these rsFC significantly contributed to 40 Hz ASSR independently in schizophrenia patients and healthy controls, while the same rsFC were also significantly reduced in schizophrenia patients.

**Table 2:** Brain areas whose functional connectivity with left or right superior temporal gyrus were significantly related to 40 Hz auditory steady-state responses.

Main effect or interaction	Seed	Peak location	BA ^a^	Coordinate	Cluster size	Peak *F*	Correlation with ASSR ^c^		HC vs SZ ^d^
				(x, y, z) ^b^			HC	SZ	*t*
ASSR	l STG	r STG	21	(59, −23, 0)	58	29.77	0.38*	0.24	3.42*
	r STG	l TPJ	39	(−50, −57, 20)	132	37.54	0.40*	0.35*	2.59*
		l MPFC	11	(−9, 45, −13)	202	26.55	0.40*	0.46*	3.20*
		l PrG	6	(−14, −17, 65)	127	27.42	0.42*	0.44*	3.38*
ASSR × Group	r STG	r Insula	-	(31, −8, 12)	58	25.57	−0.23	0.21	1.73

*ASSR* auditory steady-state responses, *HC* healthy controls, *SZ* patients with schizophrenia spectrum disorder, *STG* superior temporal gyrus, *TPJ* temporoparietal junction, *MPFC* medial prefrontal cortex, *PrG* precentral gyrus, *l* left, *r* right.

*FDR corrected *p* < 0.05 for five comparisons.

^a^Brodmann area (BA) were obtained from the TT_Daemon standard AFNI atlas [100].

^b^Talairach coordinate at peak voxel.

^c^Correlation coefficients for each group were reported here.

^d^Mean resting-state functional connectivity in each cluster was compared between the groups.

There was a significant 40 Hz ASSR and group interaction at *right STG*-right insula rsFC (Fig. 2M): this *STG*-insula rsFC was inversely correlated with 40 Hz ASSR in a healthy group but not the schizophrenia group (Fig. 2N). The correlation coefficients were significantly different between the groups (*z* = 2.61, *p* = 0.009). However, this rsFC did not show significant group differences (Fig. 2O).

Overall, rsFC in three circuits (i.e., bilateral STG rsFC and STG to MPFC and PrG rsFC) not only positively predicted 40 Hz ASSR in both groups but also were significantly weaker in patients with schizophrenia. Thus, we further used left MPFC and left PrG as seeds to explore the associations between 40 Hz ASSR and their rsFC with the rest of the brain.

### MPFC-seeded functional connectivity underlying gamma ASSR

**Fig. 3:**
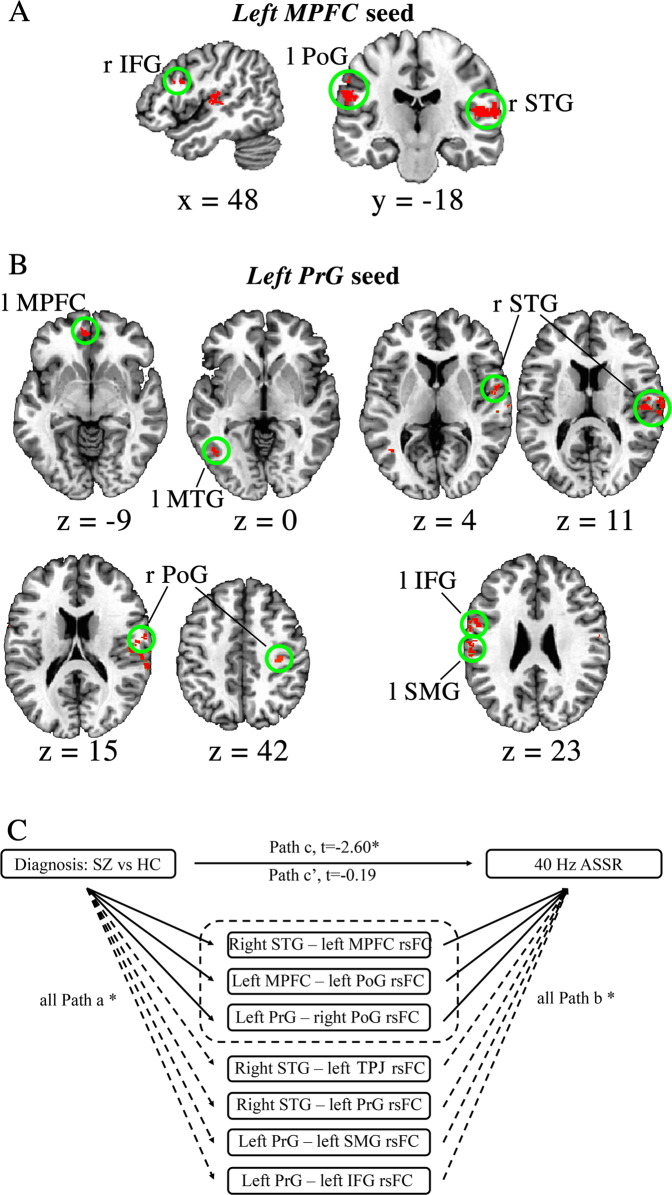
Gamma-band auditory steady-state response (ASSR) related resting-state functional connectivity (rsFC) with left medial prefrontal cortex (MPFC) or left precentral gyrus (PrG) seeds and the mediation analysis model. **A** Left *MPFC*-seeded rsFC with right STG, left postcentral gyrus (PoG), and right inferior frontal gyrus (IFG) significantly predicted 40 Hz ASSR in combined groups and within each group. **B** Left *PrG*-seeded rsFC at left supramarginal gyrus (SMG), left IFG, right PoG, left middle temporal gyrus (MTG), and left MPFC significantly predicted larger 40 Hz ASSR. Left is shown on the left side of the MRI image. **C** The significant effects of diagnosis (i.e., the differences of 40 Hz ASSR between the two diagnostic groups) on 40 Hz ASSR (path c) were nearly fully mediated by the top three rsFC (path c’ not significant). STG superior temporal gyrus, MPFC left medial prefrontal cortex, PoG postcentral gyrus, PrG precentral gyrus, SMG supramarginal gyrus, IFG inferior frontal gyrus, SZ patients with schizophrenia, HC healthy controls. Seed region was written in italic. *p < 0.05.

With left MPFC as the seed, we found significant main effects of 40 Hz ASSR at rsFC with right STG (a reciprocated finding), left postcentral gyrus (PoG), and right inferior frontal gyrus (IFG) (Fig. 3A and Table 3), with stronger rsFC predicted larger 40 Hz ASSR in the combined sample and within each group (Table 3). The correlation coefficients between the two groups were not significantly different (*z* = 0.14–1.34, *p* = 0.17–0.89). Further, schizophrenia patients had significantly reduced MPFC-seeded rsFC with right STG and left PoG (Table 3).

**Table 3:** Brain areas whose functional connectivity with the left medial prefrontal cortex or left precentral gyrus were significantly related to 40 Hz auditory steady-state responses.

Main effect or interaction	Seed	Peak location	BA ^a^	Coordinate	Cluster size	Peak *F*	Correlation with ASSR ^c^		HC vs SZ ^d^
				(x, y, z) ^b^			HC	SZ	*t*
ASSR	l MPFC	r STG	41	(54, −20, 10)	782	47.87	0.42*	0.53*	3.47*
			22	(62, −4, 5)	140	26.02	0.28*	0.40*	3.80*
		l PoG	40/2	(−59, −20, 24)	321	29.94	0.42*	0.40*	2.55*
		r IFG	9/44	(48, 12, 22)	85	28.18	0.28*	0.48*	0.38
	l PrG	r STG	41	(53, −18, 11)	314	37.41	0.44*	0.49*	4.13*
			42	(68, −11, 11)	93	21.48	0.44*	0.28*	3.55*
			22	(57, 0, 2)	85	24.14	0.39*	0.35*	2.71*
		l SMG	40	(−59, −23, 24)	162	30.19	0.35*	0.47*	3.27*
		l IFG	9	(−53, 3, 23)	154	32.90	0.34*	0.44*	3.19*
		r PoG	43	(62, −6, 16)	111	28.79	0.49*	0.42*	3.53*
			3	(33, −21, 42)	60	32.57	0.39*	0.11	0.12
		l MTG	37	(−42, −61, 0)	73	27.22	0.30*	0.45*	−0.42
		l MPFC	10	(−2, 46, −8)	55	31.04	0.43*	0.32*	1.01
ASSR × Group	l MPFC	r Insula	22	(43, −27, 0)	59	27.66	−0.21	0.51*	−0.03

*ASSR* auditory steady-state responses, *HC* healthy individuals, *SZ* patients with schizophrenia, *MPFC* medial prefrontal cortex, *STG* superior temporal gyrus, *PoG* postcentral gyrus, *IFG* inferior frontal gyrus, *PrG* precentral gyrus, *SMG* supramarginal gyrus, *MTG* middle temporal gyrus, *l* left, *r* right.

*FDR corrected *p* < 0.05 for 14 comparisons.

^a^ Brodmann area (BA) were obtained from the TT_Daemon standard AFNI atlas [100].

^b^Talairach coordinate at peak voxel.

^c^Correlation coefficients for each group were reported here.

^d^Mean resting-state functional connectivity in each cluster was compared between the groups.

There was also a significant interaction between 40 Hz ASSR and the group at left *MPFC*—right insula rsFC, which was positively associated with 40 Hz ASSR in the patient group only (Table 3). The correlation coefficients were significantly different (*z* = 4.53, *p* < 0.001), but the strength of rsFC were not significantly different between the groups (Table 3).

### PrG-seeded functional connectivity underlying gamma ASSR

With left PrG as the seed, significant main effects of 40 Hz ASSR were observed with six regions: the right STG (a reciprocated finding), left supramarginal gyrus (SMG), left IFG, right PoG, left middle temporal gyrus (MTG), and left MPFC rsFC (Fig. 3B). Stronger rsFC predicted larger 40 Hz ASSR in combined and within each group in all of these rsFC circuits (Table 3), which showed no significant difference in their correlation coefficients between the two groups *(z* = 0.27–1.76, *p* = 0.07–0.79). Schizophrenia patients exhibited significantly smaller rsFC in four of the six regions (Table 2).

In summary, we found seven rsFC circuits (nine rsFC with two reciprocated findings) were significantly associated with 40 HZ ASSR in both patients and controls and also significantly weaker in schizophrenia patients: bilateral STG, *right STG*—*left* MPFC and left PrG, *left MPFC*—left PoG, and *left PrG*—left SMG, left IFG, and right PoG rsFC, suggesting that they are directly or indirectly associated with 40 Hz ASSR independently of diagnosis or antipsychotic medications (as they were similarly observed in healthy controls) and likely are associated with 40 Hz ASSR deficit in schizophrenia (as they were also significantly reduced in schizophrenia patients).

### Mediation of gamma ASSR deficit in schizophrenia by rsFC

To further evaluate whether the above-mentioned seven rsFC mediated the 40 Hz ASSR deficit in schizophrenia, we utilized a mediation analysis method. The mediation analysis was conducted with diagnosis as the bivariate independent variable, 40 Hz ASSR as the outcome variable, rsFC as mediators, and age as a covariate. As shown in Fig. 3C, the total effect of diagnosis on 40 Hz ASSR was significant (path c; *t* = 2.69, *p* = 0.008). The direct effect from diagnosis to 40 Hz ASSR was no longer significant (path c’; *t* = −0.9, *p* = 0.93) when controlling for the rsFC mediators. Among the seven rsFC, the mediation (indirect) effects via three rsFC: *right STG*—left MPFC (95%CI = 0.15–2.66), *left MPFC*—left PoG (95%CI = 0.07–2.23), and *left PrG*—right PoG (95%CI = 0.10–3.28) rsFC were significant (Fig. 3C). When each of these three rsFC mediators was tested independently, each also showed a significant mediation on the diagnosis effects on the 40 Hz ASSR.

### Association between rsFCs and symptom and function

In patients with schizophrenia, negative symptoms as measured by BNSS were associated with the *left PrG*—right PoG rsFC (*r* = −0.27, *p* = 0.04, uncorrected). No significant correlation was found between rsFC and the severity of overall psychiatric symptoms of schizophrenia based on the BPRS total score (all |r|<0.23, *p* > 0.05) or positive symptoms based on the BPRS positive score (all |r|<0.24, *p* > 0.05). For global functioning, stronger rsFC at *left PrG*—left SMG (*r* = 0.32, *p* = 0.01, uncorrected) and *left PrG*—right PoG (*r* = 0.33, *p* = 0.01, uncorrected) predicted better global functioning in schizophrenia patients. Adding the chlorpromazine equivalent of medication dose (CPZ) as a covariate did not affect the findings.

## Discussion

The gamma band ASSR deficits in schizophrenia have been well documented, however, the aberrant neural circuits underlying reduced 40 Hz ASSR in schizophrenia remain poorly understood. Here, we found that stronger rsFC among the auditory, parietal, and prefrontal cortical regions were significantly associated with higher 40 Hz ASSR in both schizophrenia patients and controls, suggesting that these rsFC findings replicably contribute to 40 Hz ASSR across two separate samples. These associations were unlikely entirely disease-specific or due to antipsychotic medications that the patients were on, as they were similarly observed in healthy controls. Three of these rsFC circuits, i.e., *right STG*—left MPFC, *left MPFC*— left PoG, and *left PrG*—right PoG rsFC, nearly fully mediated the 40 Hz ASSR deficit in schizophrenia.

The essential role of the primary auditory cortex (i.e., STG) in gamma band ASSR generation has been established in the general population and in schizophrenia patients. We found that most of the 40 Hz ASSR-related rsFC involved right STG (Figs. 2, 3), which is in line with the findings of right lateralization of the auditory cortex for 40 Hz ASSR [74, 75]. The STG sources of 40 Hz ASSR were revealed using various source localization methods [27, 38–44]. Source localization of ASSRs in healthy individuals identified a wide range of sources both within and outside of the primary auditory cortex [43, 76, 77]. In patients with schizophrenia, interhemispheric phase locking for the primary auditory cortices was reduced in comparison to healthy controls [78]. This is consistent with the current finding that the strength of rsFC between bilateral STG (i.e., *left STG*—right STG) is positively linked to the power of 40 Hz ASSR (Table 2).

However, the study also suggested that the left medial prefrontal cortex and left precentral gyrus are also important brain areas for gamma band ASSR in schizophrenia. The role of their functional connection with STG in gamma band ASSR was first identified with STG as the seed, and then, confirmed by using MPFC or PrG as the seeds. The involvement of frontotemporal connection in 40 Hz ASSR illustrated here was in line with previous source localization evidence [43, 46, 47]. For example, a PET study found that 40-Hz ASSR activated not only primary auditory cortices but also the middle frontal gyrus;[46], and a dipole modeling to EEG data study found that sources for 40 Hz ASSR include the left frontal lobe [43]. The prefrontal and primary auditory cortices are anatomically connected [47]. Chen and others showed that besides STG, there was less activity in frontal regions during the auditory tasks in schizophrenia patients compared to healthy controls [79, 80]. Reduced gamma-band response in schizophrenia patients has been linked to impaired frontal network processing [81]. Dysfunction of the medial prefrontal cortex, which is the anterior midline node of the default mode network, in schizophrenia has been well demonstrated. Pomarol-Clotet and others used three different whole-brain voxel-based imaging techniques and identified the medial prefrontal cortex as a prominent site of abnormality in schizophrenia [82]. MPFC has also been suggested in a comparison of auditory-evoked gamma-band responses between patients with schizophrenia and healthy control subjects using an auditory reaction task [83]. They found reduced gamma band responses in schizophrenia, which was due to reduced activity in the auditory cortex and the medial frontal gyrus region. This is consistent with our findings addressing the essential role of STG and MPFC, although we used a different passive auditory task. Our data suggest that the 40 Hz ASSR deficits may be associated with reduced functional connectivity between STG and frontal areas in schizophrenia patients. i.e., the reduced *left STG*—left MPFC rsFC in schizophrenia, although the neurophysiological mechanism of this association remains unclear, in part because of the still limited understanding in the neurophysiology of rsFC.

Besides *left STG*—left MPFC rsFC, the mediation results suggest that *left MPFC*—left PoG and *left PrG*— right PoG rsFC also mediated the deficit of 40 Hz ASSR in schizophrenia. The findings with precentral and postcentral gyri may seem unusual, but precentral and postcentral gyri lesions are associated with Wernicke’s type of aphasia [84] and the interhemispheric connections of precentral and postcentral gyri were also associated with positive symptoms of schizophrenia [85, 86]. Further, decreased gray matter volumes were also observed in schizophrenia patients at bilateral precentral and left postcentral gyri [87, 88].

Previous source localization of 40 Hz ASSR in schizophrenia highlighted the role of primary auditory cortices [20, 27, 28, 34]. Our results extended this understanding by suggesting that there are non-auditory brain areas (e.g., MPFC and precentral gyrus) whose functional connections with primary auditory cortices also played roles in the gamma band ASSR deficits in schizophrenia. The mechanism underlying rsFC remained unclear, so the mechanism linking these rsFC with 40 Hz ASSR should be considered with caution, although the current data may suggest that individuals with lower baseline rsFC between these regions would have a weaker capacity to generate or sustain 40 Hz ASSR in patients and controls alike.

Deficit of ASSR is one of the robust biomarkers for schizophrenia, however, there were also studies showing null or even reversed findings [11, 31–34]. One possible reason is that ASSR powers and phase-locking values were commonly obtained from a limited number of central electrodes, e.g., FC and CZ so that rich information from other electrodes were not involved in representing ASSR responses. Here we adopted the DSS methods for evaluating ASSR power, which maximized ASSR response reliably by utilizing information from all channels, compared to the conventional ASSR power or PLV analyses that typically extract signals from a limited number of channels. The normalized ASSR power here did show a significant group difference (Fig. 1 and Table 1). However, it is worth noting that there are limitations to the 40 Hz ASSR paradigm. For instance, younger children or even young adolescents usually do not show 40 Hz ASSR as robust as adults [89–91], which limits its application in identifying at-risk individuals or the effectiveness of treatment in young first-episode patients. Moreover, deficits of low-frequency auditory responses have also been robustly observed in schizophrenia and the low-frequency activities could sometimes provide better separation between schizophrenia patients and healthy controls [32, 92–96]. The neural network origins of those low-frequency abnormalities in schizophrenia should be explored in future studies.

Other limitations of the study include that the potential effects of antipsychotic medications on current findings were unknown, although adding CPZ as an extra covariate did not significantly affect the results. The findings of similar correlation coefficients between 40 Hz ASSR and rsFC in healthy controls also suggest that these correlations are unlikely to be mainly driven by medication effects. Still, another limitation is that we used seed-based functional connectivity methods, with a limited number of seeds (i.e., left/right STG, left MPFC, and left PrG), to explore the rsFC underlying gamma band ASSR in schizophrenia. There might be other networks which do not functionally link to those seeds and also modulate 40 Hz ASSR (e.g., networks with auditory brainstem and thalamic nuclei as seeds). Conversely, it is not clear whether the rsFC identified by 40 Hz ASSR is specifically linked to 40 Hz ASSR or it is more generally associated with auditory encoding neural processes in frequencies lower than 40 Hz, as simultaneous resting fMRI and EEG recordings usually showed that, across all EEG bands, rsFC correlations with EEG are the highest at the lower frequencies [97]. Our goal here is more limited by focusing on the gamma band, which limits the specificity conclusion, although the near complete mediation of the 40 Hz effect on diagnosis by rsFC was quite surprising and encouraged future research to examine the neurophysiological interpretations for such associations. Other limiting factor is that we did not have handedness information [98, 99] on many participants so the analysis was not performed but laterality is relevant here as right hemispheric laterality for 40 Hz ASSR has been reported [74, 75].

In summary, the study explored the neural circuits underlying gamma band ASSR deficits in schizophrenia by examining the associations between ASSR and resting-state functional connectivity. We found an auditory-parietal-prefrontal network that potentially explains most of the 40 Hz ASSR deficit in schizophrenia. These findings shed light on further understanding of the mechanism of neural oscillatory deficit in schizophrenia.
